# Bonding social capital, disaster experience, and post‐disaster giving in Japan

**DOI:** 10.1111/disa.70045

**Published:** 2026-02-02

**Authors:** Toshihiro Okubo, Ilan Noy

**Affiliations:** ^1^ Keio University Japan; ^2^ Victoria University of Wellington New Zealand; ^3^ Gran Sasso Science Institute Italy

**Keywords:** earthquake, post‐disaster aid, social capital

## Abstract

When are people willing to donate their time or money after a disaster? We investigate the psychological and socio‐economic determinants of post‐disaster giving in Japan, using a nationally representative panel survey of more than 7,000 respondents, conducted repeatedly from early 2020, including after the 2024 Noto Peninsula earthquake. We examine how individual characteristics—including past disaster experience, social capital (trust, reciprocity, cooperation), ‘Big‐5’ personality traits, and digital behaviours—influence the likelihood of engaging in various forms of post‐disaster assistance, from traditional monetary donations to newer digitally‐facilitated acts such as online shopping for Noto products. Our analysis finds that prior disaster experience and personal openness are consistent robust predictors of prosocial behaviour. The relationship between social capital and aid activities is more subtle. Trust and cooperation are both positively associated with post‐disaster assistance, but this is not the case for reciprocity. These findings emphasise that nuanced conceptualisation of social capital is required and underscore the need for caution in assuming its universal relevance in mobilising disaster aid. We conclude by suggesting directions for future research that more precisely delineate the interplay between social, psychological, and socio‐economic factors in shaping post‐disaster giving.

## 1 INTRODUCTION

When are people willing to donate their time and money after a disaster? This question is clearly important, and it has been heavily researched; recent meta‐analyses of the literature on the determinants of giving include Bilén, Dreber, and Johannesson (2021) and Chapman, Hornsey, and Gillespie (2021). Some of this research involves laboratory‐ or field‐based behavioural experiments (see, for example, Andreoni, Rao, and Trachtman, 2017) or cross‐country comparisons of aggregate donations following large‐scale events (see, for example, Knowles, 2007). Most of this literature, however, uses individual‐level survey data to correlate giving decisions with donor characteristics such as gender, education, age, and other socio‐economic and demographic variables. A small subset of it examines social capital in the context of philanthropic decision making more generally. While there is a large body of work that analyses the role of social capital in shaping disaster preparedness and recovery (see, for example, Aldrich, 2012; Akbar and Aldrich, 2018; Reddy, 2023), the literature on social capital and donations in a post‐disaster assistance setting is very limited. This link between psychological traits and social capital and the willingness to donate time or money, using survey data, is the focus of this article.

The majority of the literature on the role of psychological and social capital in influencing donors' decisions assesses measures of organisational and institutional trust or community cohesiveness that concentrate on the recipients of philanthropic giving (Bekkers and Wiepking, 2011). It finds, maybe not surprisingly, that the more trustworthy and efficient a recipient is perceived to be—that is, the more recipients are trusted—the more is donated to them (irrespective of whether they are an organisation, a government, or even a country). Similarly, for philanthropic activity within a community, as community bonds are stronger and institutional trust is higher, the willingness of community members to donate to other members of one's own community is greater (see, for example, Evers and Gesthuizen, 2011; Glanville, Paxton, and Wang, 2016; Herzog and Yang, 2018).^1^

Our focus, in contrast to this literature, is on the psychological makeup of and social capital in the donors' communities; in a post‐disaster situation where the recipient community is different and far away.^2^ Specifically, we ask: what is the role of past experience of disasters, bonding social capital, measured along three dimensions (trust, reciprocity, and cooperation), and the ‘Big‐5’ psychological traits in determining the willingness of individuals to assist a far‐flung community affected by a disaster triggered by a natural hazard?

There is also literature that considers donations in the aftermath of disasters. Much of it focuses on national macroeconomic data, and typically on donating countries' cultural ties to the recipients and their geostrategic interests (see, for example, Drury, Olson, and Van Belle, 2005; Strömberg, 2007; Becerra, Cavallo, and Noy, 2015; Wei and Marinova, 2016). This literature generally does not examine micro‐level data and does not spotlight psychological traits, social cohesiveness, or social capital.

In contrast, we analyse the stated behaviour of individuals living all over Japan, in the wake of a recent disaster that hit an isolated and remote region: the Noto Peninsula earthquake in 2024. Using a large nationally representative survey, we examine statistically how the characteristics of individuals and the communities in which they live affect their willingness to assist those in the Noto region.

This is not the only earthquake to have occurred in Japan in recent times. It is, however, the deadliest event since the Great East Japan Earthquake (the triple earthquake–tsunami–nuclear disaster) struck the Tohoku region on 11 March 2011. The combined damage was enormous.^3^ Consequently, recovery required not only assistance from government but also support from the private sector and civil society and mutual aid from among residents in the affected areas. In this context, social capital within local communities has been increasingly recognised as having played a crucial part in both survival (Aldrich and Sawada, 2015) and post‐disaster recovery (Aldrich, 2011). In the stricken areas, communities with more social capital were shown to be more likely to participate in post‐disaster aid efforts (Yamauchi, 2011) and to increase the efficiency of earthquake‐related waste disposal (Kawamoto and Kim, 2016). Moreover, social capital has been shown to have reduced the risk of mental illness and psychological distress among evacuees affected by the associated nuclear accident in Fukushima (Iwagaki, Tsujiuchi, and Ogihara, 2017). Since then, the importance of social capital has been increasingly emphasised in both disaster mitigation and recovery strategies.

In response, the Japanese government has officially recognised the role of social capital in reducing disaster impacts and has promoted initiatives to foster its use as a disaster risk reduction measure.^4^ Communities with more social capital are expected to engage more actively in, for instance, volunteer activities and donating following natural hazard‐related disasters.

Meanwhile, digitalisation has advanced significantly, especially as the COVID‐19 (coronavirus disease 2019) pandemic accelerated the shift to online services (Okubo, 2022a, 2024; Baldwin and Okubo, 2024). Now, many people are accustomed to the use of digital sharing services and donate through digital applications. These multiple platforms enable donations from far afield, with little intermediation. It is all of these donation types that we correlate with the measured psychological characteristics and social capital of donors.

In the next section, we provide some background on the 2024 Noto Peninsula earthquake. Next, we describe the survey we employed, focusing specifically on post‐disaster aid activities, the donors' personal characteristics, and the measurements of social capital. We then briefly discuss the methodology, and describe our main results in detail, before providing some additional findings. The last section concludes with some caveats and ideas for future work.

## 2 BACKGROUND: THE NOTO PENINSULA EARTHQUAKE

At 4:10 PM on 1 January 2024, a large earthquake (magnitude 7.6) struck the Noto Peninsula in Ishikawa Prefecture, Japan. The disaster caused 594 deaths and more than 1,300 injuries. Approximately 193,000 buildings were damaged across nine prefectures.^5^ The earthquake also triggered a tsunami, inundating the northern coastal areas of the Peninsula. In particular, the towns of Noto, Suzu, and Wajima suffered severe damage.

The Noto Peninsula is a mountainous region with steep cliffs and an underdeveloped transportation infrastructure, consisting mainly of a few winding coastal roads. These conditions caused significant logistical difficulties following the earthquake. The main roads connecting the isolated communities were severed, and damage to the airport and seaports disrupted the delivery of supplies and the mobilisation of relief efforts. Owing to these accessibility issues, government assistance was delayed. The prolonged disruption of transport routes, the suspension of water supplies, insufficient provision of medical services, and the inefficiency of local public administrations persisted for an extended period. The delay to post‐disaster aid impeded recovery efforts and led to outmigration from the Noto region. The Peninsula's towns became increasingly isolated and depopulated. Compounding the earthquake damage, heavy rainfall struck the Noto area in September 2024, triggering landslides in many locations. These secondary events further delayed recovery.

In response, the Japanese government sought to encourage individual giving directed towards the Noto and broader Hokuriku region.^6^ In collaboration with mass media, the government promoted various forms of support beyond traditional donations, leveraging digital tools. These included online purchases of regional products from Hokuriku and the promotion of tourism in the area. The government subsidised expenses for travel to Hokuriku from other places, including hotel accommodation and dining, through digital platforms.^7^ Additionally, the promotion of ‘hometown tax’ (‘*Furusato Nozei*’) contributions to municipalities in Hokuriku was emphasised. This system, introduced in 2008, allows individuals to donate to local governments outside of their place of residence in exchange for income and residential tax credits (see, for example, Rausch, 2017; Fukasawa, Fukasawa, and Ogawa, 2020). Through this mechanism, people were encouraged to support the affected municipalities in the Noto region. These newly developed forms of aid, facilitated by digital platforms, contrasts with traditional monetary donations, which have long been customary in Japanese society.

Volunteering activities, another important type of post‐disaster giving in Japan, was also present, although initially restricted. In the aftermath of the 1995 Kobe earthquake, large numbers of ordinary people provided essential services such as food distribution, caregiving, and debris removal, significantly contributing to the city's recovery. Since then, volunteerism has become a widespread and recognised component of post‐disaster recovery in Japan. In the case of the Noto Peninsula earthquake, however, poor transportation access and the prioritisation of official relief efforts delayed the deployment of volunteers. Initially, they were not permitted to enter the affected areas, and their numbers remained limited before gradually increasing.

## 3 PANEL SURVEY AND VARIABLES TO USE

### 3.1 Panel survey data

This paper uses the Okubo–NIRA (Nippon Institute for Research Advancement) panel survey (Okubo and NIRA, 2020; Okubo, 2022a, 2022b, 2024), conducted in Japan over 13 waves (as of October 2025) and involving approximately 10,000 workers in a randomly stratified sample.^8^ The first wave was carried out in March 2020, just before the COVID‐19 pandemic spread to Japan and the declaration of the first state of emergency (in April 2020). Many respondents participated in the survey over multiple waves.^9^

The survey enquired about basic demographics (gender, age, income, and educational background), digitisation, working environments, task characteristics, occupation, place of residence, and non‐cognitive traits (such as risk attitude, social capital, and the ‘Big‐5’). Each wave added questions about a topical issue (or issues). The eleventh wave of the survey included questions about post‐disaster assistance following the Noto Peninsula earthquake (see Table A1 in the Appendix for more details about the variables collected).

One significant advantage of the panel survey used here is that many of our independent (right‐hand side) variables (on social capital, for instance) were obtained from survey waves conducted before the Noto Peninsula earthquake, so they were clearly not affected by its occurrence or by the giving decisions of people in its wake.

### 3.2 Post‐disaster assistance

We primarily utilise data from Wave 11 of the survey, which was implemented in July 2024. One of the questions asked respondents about their participation in post‐disaster assistance activities related to the Noto Peninsula earthquake. Specifically, the survey enquired about the types of aid activities respondents engaged in and the frequency of their participation.

**FIGURE 1:**
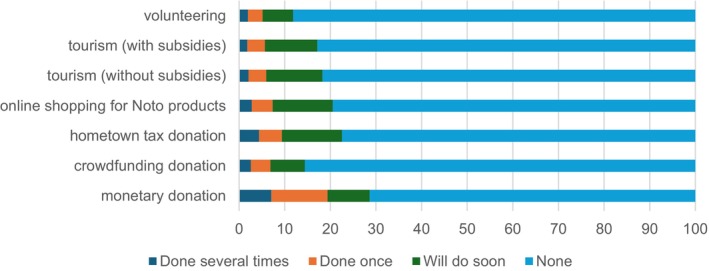
Post‐disaster assistance (percentage of 10,670 respondents). **Source:** authors.

Aid activities were divided into the following seven categories: (i) monetary donations; (ii) donating through crowdfunding; (iii) donating through hometown tax reallocation to the Hokuriku region; (iv) online shopping for Hokuriku products; (v) tourism to the Hokuriku region without government subsidies; (vi) tourism to the Hokuriku region with government subsidies; and (vii) volunteering in the region. For each activity, respondents were asked to choose one of the following options: ‘3’ for several times; ‘2’ for once; ‘1’ for not yet but plan to participate soon; or ‘0’ for never and no plan to participate. As shown in Figure 1, the percentage of respondents who either participated more than once or intended to do so soon ranged from approximately 10–30 per cent across the various aid categories. Specifically, 29 per cent participated in donations, 15 per cent in crowdfunding, 23 per cent in hometown tax contributions, 21 per cent in online shopping, 19 per cent in travel without subsidies, 18 per cent in travel with subsidies, and 12 per cent in volunteer work (see also Online Appendix 1 in the supplementary materials).^10^

Table A2 in the Appendix presents the correlation coefficients for the various assistance activities. The correlations are positive, ranging from 0.4–0.7. The highest correlation is observed between tourism with and without subsidies (r = 0.77), indicating that those who travelled without subsidies were also likely to travel with subsidies. In contrast, the correlations between monetary donations and hometown tax contributions (r = 0.38) and between monetary donations and travel without subsidies (r = 0.39) are comparatively low; these were possibly viewed as substitutes.

### 3.3 Bonding social capital

Social capital can be a crucial determinant of post‐disaster aid activities. It refers to factors like community trust, collaboration, and regional engagement that create social bonds between individuals and the community in which they live (Hanifan, 1916; Coleman, 1988; Putnam, Leonardi, and Nanetti, 1993; Bourdieu, 2023). The literature identifies three types of social capital: (i) bonding (social connections within communities); (ii) bridging (horizontal ties between communities); and (iii) linking (vertically between a community and the centre of economic, social, or political power)—these are defined in detail in Aldrich (2012). With regard to our context, we hypothesise that social capital resulted in more participation in post‐disaster assistance. The measures we use, described below, are most likely connected to both bonding and bridging social capital (that is, to generalised social capital), so we do not attempt to distinguish between them in our investigation.

Based on this hypothesis, we present three variables to measure social capital. Following the framing of Putnam, Leonardi, and Nanetti (1993), we define them as: (i) trust in other people (‘trust’); (ii) willingness and belief in mutual help (‘reciprocity’); and (iii) disposition to cooperate and contribute to one's community (‘cooperation’). In the Wave 6 questionnaire, trust in other people is measured by the average score of two statements: ‘In general, we can trust other people’ and ‘We can trust our neighbours’; reciprocity is measured by the average score of two statements: ‘People must help each other’ and ‘If you help others, they will help you when needed’; and cooperation is measured by the response to the following statement: ‘Local resources must be protected through collaboration of all local residents’. For each statement, respondents were asked to state if they strongly agree (‘5’), agree (‘4’), were neutral (‘3’), disagree (‘2’), or strongly disagree (‘1’).^11^ We note that these questions about social capital were recorded well before the Noto Peninsula earthquake, so its occurrence did not determine these measures.

### 3.4 Disaster experience

Japan is exposed to many disasters, and those with experience of past phenomena may be more likely to donate or participate in post‐disaster aid activities, since the adverse impacts of such events and the benefits of assistance are more salient to them. The Wave 10 survey, conducted in October 2023, asked respondents about their previous experience of natural hazard‐related disasters. We hypothesise that disaster experience before the Noto Peninsula earthquake could be a driver of participation in post‐disaster aid activities. We use a binary variable denoting recent previous experience of damage due to large‐scale disasters (‘1’ = experience, ‘0’ = no experience). Only 4.2 per cent of the respondents had such previous experience.^12^

### 3.5 Personality traits: the ‘Big‐5’ and attitude to risk

Willingness to provide post‐disaster assistance may also be affected by people's personalities. Following the psychological literature, we measure the Big‐5 personality traits. These refer to five standardised fundamental dimensions of personality: (i) extraversion; (ii) agreeableness; (iii) conscientiousness; (iv) neuroticism; and (v) openness (Nettle, 2009). These traits are thought to remain stable over time and are influenced by both genetics and childhood environment (Jang, Livesley, and Vernon, 1996; Soldz and Vaillant, 1999).

Extraversion highlights sociability, talkativeness, and social interest. Thus, higher extraversion means a person is more outgoing, whereas lower extraversion means a person is more reserved. Extraversion is positively associated with interest in spending more time in social contexts, and is positively predictive of alcohol consumption, popularity, parties attended, variety of dating, and exercise (Wrzus, Wagner, and Riediger, 2016). Agreeableness indicates attributes such as trust, altruism, and being kind and considerate to others. It is associated with improved ability to participate and perform well in teams (Bell, 2007; Bradley et al., 2013). Openness features characteristics such as creativity, imagination, and insight; and it signifies a broad range of interests. It is positively associated with interacting with strangers and negatively associated with doing nothing (Wrzus, Wagner, and Riediger, 2016). Conscientiousness relates to thoughtfulness and goal‐directed behaviours. It is associated with behaving responsibly, carefully, and with self‐discipline. Neuroticism is characterised by frequent worries, sadness, and emotional instability (Mehl, Gosling, and Pennebaker, 2006).

Lastly, regarding attitude to risk, the survey asked respondents to choose a number from ‘0’ to ‘10’ to indicate their risk preference, where ‘0’ means ‘completely unwilling to take risks’ and ‘10’ means ‘very willing to take risks’.

## 4 ESTIMATIONS AND RESULTS

### 4.1 Post‐disaster aid activities and individual factors

We sought to investigate how individual characteristics, in particular social capital, affect post‐disaster activities. Using ordered logit, we posit that:

(1) $$\text{prob}\left({\mathrm{Aid}}_{i}\right)=\phi \left({\mathrm{\beta X}}_{i}+{\mathrm{\gamma Z}}_{i}+\varepsilon_{i}\right)$$

For the dependent variable, ${\mathrm{Aid}}_{i}$ is the frequency of each aid behaviour: never and no plan (‘0’); not yet but have a plan to do soon (‘1’); once (‘2’); or several times (‘3’). As mentioned above, aid or assistance activity is composed of seven possible actions, ranging from monetary donations to volunteering in person.

$X_{i}$ is the set of variables on which we focus as determinants of the decision to participate in a giving activity. These include measures of bonding social capital, disaster experience, risk attitude, and the Big‐5 psychological traits. $Z_{i}$ is a vector of demographic and personal traits (gender, age, income, and education), including occupation and prefecture fixed effects. We note that gender (female = ‘2’, male = ‘1’), age (scaled by age 10), income (annual income of 2023 scaled by JPY 500,000), education (junior high school = ‘1’, high school = ‘2’, college/vocational school = ‘3’, university (undergraduate) = ‘4’, master's degree = ‘5’, and doctoral degree = ‘6’). Partial regression results are reported in Table 1; the full results are available in Online Appendix 2 in the supplementary materials.

**TABLE 1:** Ordered‐logit regression results, benchmark specification.

	Donation	Crowdfunding donations	Hometown donations to Hokuriku	Online shopping for Hokuriku products	Tourism in Hokuriku (without subsidies)	Tourism in Hokuriku (with subsidies)	Volunteer activities
Past disaster experience	1.04***	1.62***	1.37***	1.39***	1.50***	1.46***	1.64***
	(9.96)	(13.67)	(11.56)	(9.50)	(9.27)	(9.53)	(9.87)
Big‐5 openness	0.12***	0.24***	0.11***	0.16***	0.09**	0.17***	0.22***
	(4.53)	(6.34)	(4.01)	(7.57)	(2.45)	(3.71)	(5.48)
Trust	0.02	0.20***	0.19***	0.11**	0.08	0.07	0.24***
	(0.58)	(2.82)	(3.44)	(2.56)	(1.39)	(1.22)	(4.12)
Reciprocity	0.18***	−0.20***	−0.01	−0.01	−0.01	−0.06	−0.20***
	(4.88)	(−3.32)	(−0.10)	(−0.14)	(−0.13)	(−0.71)	(−3.33)
Cooperation	0.30***	0.02	0.10**	0.24***	0.12**	0.13**	−0.18***
	(6.60)	(0.28)	(2.20)	(6.15)	(2.37)	(2.34)	(−3.81)
Log pseudo‐likelihood	−6,081.68	−3,500.76	−4,982.82	−4,571.24	−4,052.89	−3,866.31	−2,825.59

**Notes:** all regressions were estimated using an ordered‐logit model, with standard errors clustered at the prefecture level. All include 7,194 observations. Additional control variables not reported in the table but included in the regressions were: age; gender; education; income; risk attitude; and the four unreported Big‐5 personality traits.

**Source:** authors.

Gender is statistically significant for three of the seven regressions, consistently showing that females are more likely to engage in assistance. Income is also consistently positive, and statistically significant in three of the seven regressions. Age is consistently negative and significant for all assistance activities, except for the most traditional one (monetary donations). The risk and education measures are consistently positive and statistically significant in all seven regressions. The Big‐5 personality traits are not consistently significant—in a few cases they are weakly significant at the 10 per cent level—except for the measure of openness. The latter appears to be a clear positive predictor of donation behaviour, across the different assistance measures we analyse, so we have included it in Table 1.^13^

Consistently, we find that having experienced a disaster in the recent past increases the likelihood that a person will provide aid to the Noto communities. The magnitude of the coefficient is similar across all activities except for traditional monetary donations, where the connection between recent experience of disaster and current philanthropic activity is weaker.

For the social capital variables, the picture is more nuanced. Trust in others is consistently positive (that is, people who trust others are more likely to engage in mutual aid), although this result is not always statistically significant. Reciprocity, the idea that people rely on each other in reciprocal relationships of give and take, is not consistently statistically significant and is often negative. One potential explanation is that, in this case, the donors are far away from the recipients; hence, this transactional approach is less conducive to behaviour that relies on giving to distant anonymous people and that will not be echoed. The measure of cooperation is, once again, consistently positive and statistically significant, except for a negative coefficient for people who chose to volunteer their time/work.

As a robustness check, we estimated the same regressions using a logit estimation, instead of the ordered‐logit model shown in Table 1. The giving variables were transformed into binary measures, with ‘0’ indicating no intention to donate time or money and ‘1’ representing all of the other options (from once to several times, to having a concrete plan to give). These results are qualitatively identical to the ordered‐logit results and are available in Online Appendix 3 in the supplementary materials.

## 5 FURTHER INVESTIGATIONS

We conducted more estimations to achieve a more detailed and robust analysis. First, we wanted to see if our previous results are sensitive to the inclusion of measures that are a proxy for willingness to engage in long‐distance donating activities, which are possibly conditional on the degree of digital understanding. For that purpose, we added four additional variables to the estimated regression: (i) whether people mostly use digital payments; (ii) whether they mostly use cash; (iii) whether they engage in the sharing economy (or the platform economy); and (iv) whether they shop online.^14^ See Table 2 for the results; see also Online Appendix 4 in the supplementary materials.

**TABLE 2:** Ordered‐logit regression results, specification with digitalisation measures.

	Donation	Crowdfunding donations	Hometown donations to Hokuriku	Online shopping for Hokuriku products	Tourism in Hokuriku (without subsidies)	Tourism in Hokuriku (with subsidies)	Volunteer activities
Past disaster experience	0.80***	1.24***	1.08***	1.09***	1.16***	1.07***	1.19***
	(7.36)	(12.01)	(10.16)	(8.56)	(7.39)	(7.37)	(7.19)
Big‐5 openness	0.09***	0.20***	0.07**	0.11***	0.04	0.12**	0.19***
	(3.49)	(5.12)	(2.36)	(4.80)	(1.06)	(2.57)	(4.17)
Trust	0.01	0.17**	0.18***	0.09*	0.07	0.04	0.23***
	(0.35)	(2.25)	(3.03)	(1.94)	(1.10)	(0.65)	(4.05)
Reciprocity	0.17***	−0.17**	0.00	0.00	−0.01	−0.03	−0.14**
	(4.49)	(−2.33)	(0.04)	(−0.06)	(−0.08)	(−0.36)	(−2.30)
Cooperation	0.28***	0.02	0.08	0.24***	0.11**	0.14**	−0.18***
	(6.30)	(0.25)	(1.54)	(4.98)	(1.99)	(1.97)	(−3.67)
Digital payments	0.28***	0.13*	0.40***	0.17***	0.32***	0.28***	0.01
	(4.16)	(1.91)	(7.07)	(3.64)	(5.58)	(5.31)	(0.26)
Cash payments	0.13***	−0.12***	−0.09**	−0.07**	−0.03	−0.12***	−0.13***
	(3.82)	(−3.22)	(−2.44)	(−2.20)	(−0.99)	(−2.94)	(−2.87)
Sharing economy	0.41***	0.78***	0.55***	0.65***	0.71***	0.83***	0.90***
	(8.64)	(11.11)	(8.39)	(8.40)	(12.48)	(11.54)	(14.06)
Online shopping	0.40***	0.49***	0.37***	0.60***	0.35***	0.32***	0.35***
	(9.85)	(7.80)	(6.65)	(9.18)	(5.36)	(5.10)	(4.85)
Log pseudo‐likelihood	−5,864.36	−3,224.36	−4,713.82	−4,255.45	−3,780.68	−3,559.68	−2,594.91

**Note:** all regressions were estimated using an ordered‐logit model, with standard errors clustered at the prefecture level. All include 7,194 observations. Additional control variables not reported in the table but included in the regressions were: age; gender; education; income; risk attitude; and the four unreported Big‐5 personality traits.

**Source:** authors.

Generally, the possibly unsurprising picture that emerges from this analysis is that people who are more comfortable with digital payments, the sharing economy, and online shopping are also more likely to engage in all seven types of donating activities and volunteering that we examine. Equally, using cash is less associated with all of these activities, except for traditional monetary donations. The previously identified positive relationship between past experience of disasters and a current willingness to donate is maintained, as is the positive association with openness (Big‐5).

The previously identified positive association with the trust and cooperation dimensions of social capital is robust to the inclusion of these digital economy variables. Reciprocity, as reported in Table 1 as well, is mostly statistically insignificant. Overall, we can conclude that the identified relationship between social capital and giving behaviour is robust to the inclusion of these digitalisation measures, which as expected are statistically significant themselves.

One may expect people's work environment to influence aid behaviour. De la Rica and Gortazar (2016) constructed measures for work‐related tasks by using the PIAAC (Programme for the International Assessment of Adult Competencies) questionnaire (see Table A3 in the Appendix). Tasks for each individual are divided into three components: (i) ‘manual’; (ii) ‘abstract’; and (iii) ‘routine’. Therein, ‘manual’ is defined as physical tasks, ‘abstract’ is defined as tasks mostly involving cognitive and interpersonal non‐repetitive tasks, and ‘routine’ is defined as repetitive cognitive and/or manual tasks. The indexes for each of them are derived from the first component of a principal component analysis and then standardised. We hypothesise that individuals engaged in mostly manual tasks may be more likely or more inclined to participate in post‐disaster volunteer work (which typically involves physical labour)—the results are reported in Table 3; for full results, see Online Appendix 5 in the supplementary materials. Yet, we find little in the way of a pattern that differentiates between the different time and monetary donation types. Overall, people whose time is mostly devoted to manual tasks or to abstract tasks are more likely to pursue most of the kinds of philanthropy we analysed.

**TABLE 3:** Ordered‐logit regression results, specification with work‐type measures.

	Donation	Crowdfunding donations	Hometown donations to Hokuriku	Online shopping for Hokuriku products	Tourism in Hokuriku (without subsidies)	Tourism in Hokuriku (with subsidies)	Volunteer activities
Past disaster experience	0.94***	1.49***	1.26***	1.28***	1.39***	1.35***	1.49***
	(9.30)	(13.17)	(10.96)	(9.35)	(8.86)	(9.01)	(9.42)
Big‐5 openness	0.10***	0.21***	0.08***	0.13***	0.06	0.14***	0.20***
	(4.11)	(5.80)	(3.09)	(6.10)	(1.62)	(3.14)	(4.40)
Trust	0.03	0.21***	0.21***	0.12***	0.10*	0.09	0.26***
	(0.74)	(2.97)	(3.67)	(2.71)	(1.67)	(1.54)	(4.50)
Reciprocity	0.15***	−0.24***	−0.04	−0.04	−0.05	−0.10	−0.23***
	(3.98)	(−3.87)	(−0.68)	(−0.71)	(−0.76)	(−1.17)	(−3.94)
Cooperation	0.28***	0.01	0.08*	0.22***	0.10**	0.11**	−0.19***
	(6.33)	(0.09)	(1.77)	(5.94)	(2.11)	(2.21)	(−3.94)
Routine	−0.09**	−0.11**	−0.09**	−0.20***	−0.12***	−0.11**	−0.01
	(−2.57)	(−2.39)	(−2.15)	(−4.37)	(−2.58)	(−2.50)	(−0.17)
Abstract	0.25***	0.23***	0.31***	0.21***	0.26***	0.28***	0.32***
	(6.33)	(4.35)	(7.40)	(5.37)	(5.36)	(5.99)	(6.53)
Manual	0.11***	0.20***	0.07*	0.11***	0.12***	0.15***	0.23***
	(2.85)	(4.39)	(1.75)	(2.98)	(4.17)	(3.98)	(4.48)
Log pseudo‐likelihood	−6004.9	−3435.94	−4908.07	−4488.9	−3984.48	−3790.58	−2767.28

**Note:** all regressions were estimated using an ordered‐logit model, with standard errors clustered at the prefecture level. All include 7,194 observations. Additional control variables not reported in the table but included in the regressions were: age; gender; education; income; risk attitude; and the four unreported Big‐5 personality traits.

**Source:** authors.

The ‘past disaster experience’ variable is positive and significant, and the openness measure is as well. Both are similar to the benchmark regression results described in Table 1. The statistical significance and the size of the coefficient of the social capital variables (trust, reciprocity, and cooperation) do not change by much once controls for the work environment are included in the specifications.

In addition to the above about people's working environment, the survey includes several questions related to workplace settings (see the Appendix for details). In particular, it asks about teamwork‐based workplaces, the presence of a flexitime system, and performance‐based wages. The results indicate that individuals with access to flexible worktime arrangements and to performance‐based wages are more likely to engage in all types of volunteering and giving activities. Similar to the analysis of the work‐task characterisations we analysed, the statistical significance of the social capital measures, past experience of disasters, and the Big‐5 openness measure are all robust to the inclusion of these additional controls. These results are available in Online Appendix 6 in the supplementary materials.

Geographical proximity may also affect willingness to participate in post‐disaster aid activities. To examine this, we calculated the geographical distance between Wajima City (a central location in the Noto region) to the prefectural capital of each respondent's place of residence (we only know the prefecture of residence). We hypothesised that the closer a respondent lives to the affected area, the more likely they are to engage in aid‐related activities for which distance matters, such as tourism and volunteer work (see, for example, Yamamura, 2013; Nissen, Carlton, and Wong, 2022). This is indeed what we found, with the coefficient on the distance measure negative and significant only for these types of assistance. Once again, the results with respect to social capital are robust—see Online Appendix 7 in the supplementary materials.

## 6 CONCLUSION

We examined the determinants of post‐disaster assistance choices in Japan following the 2024 Noto Peninsula earthquake, using a nationally representative survey. The analysis focused on the role of psychological traits, bonding social capital, individual past disaster experiences, and other measures of people's familiarity with digital technologies and work environments. Using these data, we found consistent associations between willingness to give, through various philanthropic channels, and past disaster exposure and the Big‐5 openness personality trait. Our analysis also revealed a robust connection between traditional measures of bonding and bridging social capital and post‐disaster giving. Specifically, trust and cooperation are both positively associated with philanthropic acts, while reciprocity is mostly unrelated. These effects are not sensitive to model specification, and to the inclusion of controls for digital behaviour, work environment, and geographical proximity.

We found that social capital matters to individuals engaging in a wide range of philanthropic activities. As such, this suggests another avenue for public policy that attempts to improve bonding social capital. Increasing trust, and civic engagement (cooperation), were already seen to reduce disaster damage and loss and benefit post‐disaster recovery. But it now appears that they are also conducive in relation to mutual assistance and the philanthropic involvement of external sources during the recovery process.

Future research should further disentangle these overlapping influences. In particular, clearer classifications of giving behaviour, and a more distinct differentiation of measures of bonding and bridging social capital, could further help to conceptualise more precisely the connections we have described, as well as reveal their robustness to different cultural and geographical settings. Experimental research designs at this nexus can also possibly clarify how public policy can create or generate the conditions for social capital to facilitate increases in philanthropic giving behaviour in the aftermath of disasters.

## CONFLICT OF INTEREST STATEMENT

Both authors declare no conflict of interest.

## Supporting information


**Appendix S1:** Supporting Information.

## Data Availability

The data that support the findings of this study are available on request from the corresponding author. The data are not publicly available due to privacy or ethical restrictions.

## Endnotes

^1^
Some of these papers do not identify the recipients and appraise the general philanthropic behaviour of donors. We assume most of this refers, typically, to local donations as local philanthropy is substantially more common (see, for example, Robson and Hart, 2020).

^2^
An exception to this focus on recipients' social capital is Knowles (2007), who uses macro‐level national data on social capital to compare the foreign aid generosity of different countries.

^3^
It was the costliest event globally, since 1900, as recorded by the Emergency Events Database (EM‐DAT)—for more information, see https://www.emdat.be/ (last accessed on 20 January 2026).

^4^
Similar initiatives have been introduced in other countries as well.

^5^
Most of the damage was by far in the Noto Peninsula. The Japanese Cabinet Office provides reports on damage, recovery, and post‐disaster aid activities once every few months. For more information, see https://www.bousai.go.jp/updates/r60101notojishin/r60101notojishin/index.html (last accessed on 20 January 2026).

^6^
The Hokuriku region refers to Ishikawa Prefecture as well as four neighbouring prefectures.

^7^
This type of subsidy programme was already implemented during the COVID‐19 pandemic, named the ‘Go to Travel Campaign’ (for details, see Okubo, 2022b).

^8^
For the data link, see https://nira.or.jp/paper/data/2022/26.html (last accessed on 20 January 2026).

^9^
The survey was conducted on a website constructed by Nikkei Research. It employs a stratified random sampling strategy: Japan is stratified into five regions by regional classification and six age groups for each gender (12 age–gender groups per region). As such, the sample was stratified into 60 demographic groups whose sizes, when sampled, were based on each group's share of the general population. The Japan Labour Force Survey by the Ministry of Internal Affairs and Telecommunications served as the sampling unit. The survey sought a panel structure and thus to keep the same respondents over waves as much as possible. Some respondents repeatedly participated in the survey. New respondents were added to meet the required sample sizes (for more details, see Okubo, 2022a).

^10^
All supplementary materials can be found online in the supporting information section at the end of the article.

^11^
Our social capital framework is very similar to that of Yodo (2018) who defined the three dimensions of bonding social capital similarly; see also Okubo and Noy (2025). Other comparable frameworks applied in the Japanese context can be found in Kawachi, Kennedy, and Glass (1999).

^12^
This is a relatively small percentage because the survey asked about experience during a short recent period, from May–October 2023. These data are shown in Table A1 in the Appendix.

^13^
All results are posted in the online supplementary materials.

^14^
In the questionnaire, respondents were asked about the frequency of each digital service in their daily life (frequent = ‘3’, occasional = ‘2’, seldom = ‘1’, and none = ‘0’). The items are (i) credit card payment, (ii) debit card payment, (iii) pre‐paid electric money payment, (iv) fintech (financial technology) services, (v) internet banking, (vi) cash payment, (vii) internet delivery services, (viii) online shopping, and (ix) sharing services. We categorised (i)–(v) as digital payment, (vi) as cash payment, (vii) and (viii) as online delivery shopping, and (ix) as sharing economy service. Then we took an average for each category. These questions were included in Wave 11.

## APPENDIX A

### A.1

**TABLE A1:** Variables.

	Variables	Mean	Standard deviation	Minimum	Maximum
Post‐earthquake assistance	Donation	0.53	0.94	0	3
	Crowdfunding donation	0.22	0.63	0	3
	Hometown donation	0.36	0.77	0	3
	Online shopping	0.30	0.67	0	3
	Tourism in Hokuriku (without subsidies)	0.25	0.61	0	3
	Tourism in Hokuriku (with subsidies)	0.23	0.59	0	3
	Volunteer activities	0.17	0.54	0	3
Personal characteristics	Gender (female = ‘2’, male = ‘1’)	1.44	0.50	1	2
	Income	4.14	3.64	0.25	21.25
	Age	8.33	2.64	2	12
	Risk	3.92	2.24	0	10
	Education	3.32	1.01	1	6
	Past disaster experience	0.04	0.20	0	1
Big‐5 personality traits	Extraversion	3.72	1.21	1	7
	Agreeableness	4.07	1.21	1	7
	Conscientiousness	4.10	1.09	1	7
	Neuroticism	3.93	1.10	1	7
	Openness	3.79	1.07	1	7
Social capital	Trust	3.00	0.86	1	5
	Reciprocity	3.44	0.80	1	5
	Cooperation	3.31	0.85	1	5
Other variables	Digital payments	1.27	0.65	0	3
	Cash payments	2.03	0.92	0	3
	Sharing economy	0.26	0.63	0	3
	Online shopping	1.04	0.69	0	3
	Teamwork base	0.31	0.98	−2	2
	Outcome base wage	−0.20	0.90	−2	2
	Flexible time	−0.09	0.97	−2	2
	Log distance from Noto	5.87	0.43	4.41	7.32

**Note:** the sample contains 7,194 observations.

**Source:** authors.

**TABLE A2:** Correlations between assistance variables.

	Donation	Crowdfunding	Hometown tax	Online shopping	Tourism (without subsidies)	Tourism (with subsidies)	Volunteer
Donation	1						
Crowdfunding	0.4853	1					
Hometown tax	0.3873	0.5907	1				
Online shopping	0.4474	0.6173	0.5975	1			
Tourism (without subsidies)	0.3948	0.6202	0.5576	0.6696	1		
Tourism (with subsidies)	0.405	0.66	0.5707	0.6664	0.7696	1	
Volunteer	0.4028	0.7005	0.5575	0.6077	0.6962	0.7448	1

**Source:** authors.

**TABLE A3:** Task question items from the PIAAC questionnaire (De la Rica and Gortazar, 2016).

Task	Category	Items	PIAAC Item No.
Abstract (non‐routine)	Cognitive interpersonal and non‐routine	Read diagrams, maps, or schematics	G_Q01h
		Write reports	G_Q02c
		Face complex problems	F_Q05b
		Persuade, influence people	F_Q04a
		Negotiate with people	F_Q04b
Routine	Cognitive routine	Change task sequence	D_Q11a
		Change how work is done	D_Q11b
		Change speed of work	D_Q11c
		Change working hours	D_Q11d
		Learn work‐related things from coworkers	D_Q13a
		Learn by doing, based on tasks performed	D_Q11d
		Keep up to date on new products/services	D_Q13c
	Manual routine	Hand/finger skill accuracy	F_Q06c
Manual		Physical work	F_Q06b

**Source:** authors.

**Construction of variables for the working environment**

Our survey asks about working environments in relation to six items pertaining to team collaboration, outcome‐based evaluation, and flexible employment systems:

1. The tasks under your charge are clearly specified within the team.
2. The team cooperates on your tasks.
3. Your workplace evaluates working hard irrespective of working hours.
4. Your job evaluation is based on outcome.
5. You have the flexibility to choose working hours and places.
6. You can easily take leave for family reasons (taking care of children and nursing elderly persons).

For each item, a respondent chooses either disagree (‘‐2’ for our calculation), weakly disagree (‘‐1’), neutral (‘0’), weakly agree (‘1’), or agree (‘2’). To construct variables for working environments, we sum up to three categories, characterised by team‐based work (mean of items 1 and 2), outcome‐based wage (mean of items 3 and 4), and flexitime (mean of items 5 and 6).

## References

[disa70045-bib-0001] Akbar, M.S. and D.P. Aldrich (2018) ‘Social capital's role in recovery: evidence from communities affected by the 2010 Pakistan floods’. Disasters. 42(3). pp. 475–497. 29131374 10.1111/disa.12259

[disa70045-bib-0002] Aldrich, D.P. (2011) ‘The power of people: social capital's role in recovery from the 1995 Kobe earthquake’. Natural Hazards. 56(3). pp. 595–611.

[disa70045-bib-0003] Aldrich, D.P. (2012) ‘Social, not physical, infrastructure: the critical role of civil society after the 1923 Tokyo earthquake’. Disasters. 36(3). pp. 398–419. 22066778 10.1111/j.1467-7717.2011.01263.x

[disa70045-bib-0004] Aldrich, D.P. and Y. Sawada (2015) ‘The physical and social determinants of mortality in the 3.11 tsunami’. Social Science & Medicine. 124(January). pp. 66–75. 25461863 10.1016/j.socscimed.2014.11.025

[disa70045-bib-0005] Andreoni, J. , J.M. Rao , and H. Trachtman (2017) ‘Avoiding the ask: a field experiment on altruism, empathy, and charitable giving’. Journal of Political Economy. 125(3). pp. 625–653.

[disa70045-bib-0006] Baldwin, R. and T. Okubo (2024) ‘Are software automation and teleworker substitutes? Preliminary evidence from Japan’. The World Economy. 47(4). pp. 1531–1556.

[disa70045-bib-0007] Becerra, O. , E. Cavallo , and I. Noy (2015) ‘Where is the money? Post‐disaster foreign aid flows’. Environment and Development Economics. 20(5). pp. 561–586.

[disa70045-bib-0008] Bekkers, R. and P. Wiepking (2011) ‘A literature review of empirical studies of philanthropy: eight mechanisms that drive charitable giving’. Nonprofit and Voluntary Sector Quarterly. 40(5). pp. 924–973.

[disa70045-bib-0009] Bell, S.T. (2007) ‘Deep‐level composition variables as predictors of team performance: a meta‐analysis’. Journal of Applied Psychology. 92(3). pp. 595–615. 17484544 10.1037/0021-9010.92.3.595

[disa70045-bib-0010] Bilén, D. , A. Dreber , and M. Johannesson (2021) ‘Are women more generous than men? A meta‐analysis’. Journal of the Economic Science Association. 7(1). pp. 1–18.

[disa70045-bib-0011] Bourdieu, P. (2023) ‘The forms of capital 1’. In W. Longhofer and D. Winchester (eds.) Social Theory Re‐Wired: New Connections to Classical and Contemporary Perspectives. Third edition. Routledge, New York City, NY. pp. 165–176.

[disa70045-bib-0012] Bradley, B.H. , J.E. Baur , C.G. Banford , and B.E. Postlethwaite (2013) ‘Team players and collective performance: how agreeableness affects team performance over time’. Small Group Research. 44(6). pp. 680–711.

[disa70045-bib-0013] Chapman, C.M. , M.J. Hornsey , and N. Gillespie (2021) ‘To what extent is trust a prerequisite for charitable giving? A systematic review and meta‐analysis’. Nonprofit and Voluntary Sector Quarterly. 50(6). pp. 1274–1303.

[disa70045-bib-0014] Coleman, J.S. (1988) ‘Social capital in the creation of human capital’. American Journal of Sociology. 94(S1). pp. S95–S120.

[disa70045-bib-0015] De La Rica, S. and L. Gortazar (2016) *Differences in Job De‐Routinization in OECD Countries: Evidence from PIAAC*. IZA Discussion Paper. IZA DP No. 9736. February. IZA – Institute of Labor Economics, Bonn.

[disa70045-bib-0016] Drury, A.C. , R.S. Olson , and D.A. Van Belle (2005) ‘The politics of humanitarian aid: U.S. foreign disaster assistance, 1964–1995’. The Journal of Politics. 67(2). pp. 454–473.

[disa70045-bib-0017] Evers, A. and M. Gesthuizen (2011) ‘The impact of generalized and institutional trust on donating to activist, leisure, and interest organizations: individual and contextual effects’. International Journal of Nonprofit and Voluntary Sector Marketing. 16(4). pp. 381–392.

[disa70045-bib-0018] Fukasawa, E. , T. Fukasawa , and H. Ogawa (2020) ‘Intergovernmental competition for donations: the case of the Furusato Nozei program in Japan’. Journal of Asian Economics. 67(3). Article number: 101178. 10.1016/j.asieco.2020.101178.

[disa70045-bib-0019] Glanville, J.L. , P. Paxton , and Y. Wang (2016) ‘Social capital and generosity: a multilevel analysis’. Nonprofit and Voluntary Sector Quarterly. 45(3). pp. 526–547.

[disa70045-bib-0020] Hanifan, L.J. (1916) ‘The rural school community center’. The Annals of the American Academy of Political and Social Science. 67(September). pp. 130–138.

[disa70045-bib-0021] Herzog, P.S. and S. Yang (2018) ‘Social networks and charitable giving: trusting, doing, asking, and alter primacy’. Nonprofit and Voluntary Sector Quarterly. 47(2). pp. 376–394.

[disa70045-bib-0022] Iwagaki, T. , T. Tsujiuchi , and A. Ogihara (2017) ‘Social capital and mental health in a major disaster: findings and suggestions from the survey and social support after the Fukushima nuclear disaster’. Shinshin Igaku. 57(10). pp. 1013–1019.

[disa70045-bib-0023] Jang, K.L. , W.J. Livesley , and P.A. Vernon (1996) ‘Heritability of the Big Five personality dimensions and their facets: a twin study’. Journal of Personality. 64(3). pp. 577–592. 8776880 10.1111/j.1467-6494.1996.tb00522.x

[disa70045-bib-0024] Kawachi, I. , B.P. Kennedy , and R. Glass (1999) ‘Social capital and self‐rated health: a contextual analysis’. American Journal of Public Health. 89(8). pp. 1187–1193. 10432904 10.2105/ajph.89.8.1187 PMC1508687

[disa70045-bib-0025] Kawamoto, K. and K. Kim (2016) ‘Social capital and efficiency of earthquake waste management in Japan’. International Journal of Disaster Risk Reduction. 18(September). pp. 256–266.

[disa70045-bib-0026] Knowles, S. (2007) ‘Social capital, egalitarianism and foreign aid allocations’. Journal of International Development. 19(3). pp. 299–314.

[disa70045-bib-0027] Mehl, M.R. , S.D. Gosling , and J.W. Pennebaker (2006) ‘Personality in its natural habitat: manifestations and implicit folk theories of personality in daily life’. Journal of Personality and Social Psychology. 90(5). pp. 862–877. 16737378 10.1037/0022-3514.90.5.862

[disa70045-bib-0028] Nettle, D. (2009) Personality: What Makes You the Way You Are. Oxford University Press, Oxford.

[disa70045-bib-0029] Nissen, S. , S. Carlton , and J.H.K. Wong (2022) ‘Gaining “authority to operate”: student‐led emergent volunteers and established response agencies in the Canterbury earthquakes’. Disasters. 46(3). pp. 832–852. 34120355 10.1111/disa.12496

[disa70045-bib-0030] Okubo, T. (2022a) ‘Telework in the spread of COVID‐19’. Information Economics and Policy. 60(September). Article number: 100987. 10.1016/j.infoecopol.2022.100987.

[disa70045-bib-0031] Okubo, T. (2022b) ‘Traveling and eating out during the COVID‐19 pandemic: the Go To campaign policies in Japan’. Japan and the World Economy. 64(December). Article number: 101157. 10.1016/j.japwor.2022.101157. 36157374 PMC9482085

[disa70045-bib-0032] Okubo, T. (2024) ‘Non‐routine tasks and ICT tools in telework’. Labour. 38(2). pp. 177–202.

[disa70045-bib-0033] Okubo, T. and NIRA (Nippon Institute for Research Advancement) (2020) Report on the Results of a Questionnaire Survey Concerning the Impact of the Use of Telework to Respond to the Spread of COVID‐19 on Working Styles, Lifestyles, and Awareness. NIRA, Tokyo.

[disa70045-bib-0034] Okubo, T. and I. Noy (2025) ‘Vaccination decisions and social capital in Japan’. SSM – Population Health. 30(June). Article number: 101769. 10.1016/j.ssmph.2025.101769. 40124530 PMC11925093

[disa70045-bib-0035] Putnam, R.D. , R. Leonardi , and R.Y. Nanetti (1993) Making Democracy Work: Civic Traditions in Modern Italy. Princeton University Press, Princeton, NJ.

[disa70045-bib-0036] Rausch, A. (2017) ‘Japan's Furusato Nozei tax system: shared Japanese citizenship or rewarded local place appeal?’. Electronic Journal of Contemporary Japanese Studies. 17(1). https://www.japanesestudies.org.uk/ejcjs/vol17/iss1/rausch.html (last accessed on 21 January 2026).

[disa70045-bib-0037] Reddy, M. (2023) ‘Flattening the curve: voluntary association participation and the 2013–16 West Africa Ebola epidemic’. Disasters. 47(2). pp. 366–388. 35612956 10.1111/disa.12548

[disa70045-bib-0038] Robson, A. and D.J. Hart (2020) ‘Understanding the correlates of donor intention: a comparison of local, national, and international charity destinations’. Nonprofit and Voluntary Sector Quarterly. 50(3). pp. 506–530.

[disa70045-bib-0039] Soldz, S. and G.E. Vaillant (1999) ‘The Big Five personality traits and the life course: a 45‐year longitudinal study’. Journal of Research in Personality. 33(2). pp. 208–232.

[disa70045-bib-0040] Strömberg, D. (2007) ‘Natural disasters, economic development, and humanitarian aid’. Journal of Economic Perspectives. 21(3). pp. 199–222.

[disa70045-bib-0041] Wei, J. and D. Marinova (2016) ‘The orientation of disaster donations: differences in the global response to five major earthquakes’. Disasters. 40(3). pp. 452–475. 27295360 10.1111/disa.12160

[disa70045-bib-0042] Wrzus, C. , G.G. Wagner , and M. Riediger (2016) ‘Personality–situation transactions from adolescence to old age’. Journal of Personality and Social Psychology. 110(5). pp. 782–799. 26167797 10.1037/pspp0000054

[disa70045-bib-0043] Yamamura, E. (2013) ‘Natural disasters and participation in volunteer activities: a case study of the Great Hanshin‐Awaji Earthquake’. Annals of Public and Cooperative Economics. 84(1). pp. 103–117.

[disa70045-bib-0044] Yamauchi, N. (2011) Bousai, Saigai Fukko ni okeru Social Capital no Yakuwari (Role of Social Capital in Disaster Prevention and Recovery). Report. Research Institute for Advancement of Living Standards, Tokyo.

[disa70045-bib-0045] Yodo, M. (2018) So‐sharu Kyapitaru no Keizai Bunseki (Economic Analysis on Social Capital). Keio University Press, Tokyo.

