# Supplementary material for: Bonding social capital, disaster experience, and post‐disaster giving in Japan
Source: Disasters. 2026 Feb 2;50(2):e70045. doi: 10.1111/disa.70045 (PMC12865337; doi:10.1111/disa.70045)
Supplement: Supplementary file 1 — Appendix S1: Supporting Information. [file DISA-50-e70045-s001.docx]

**Bonding social capital, disaster experience, and post-disaster giving in Japan**

**ONLINE SUPPLEMENTARY MATERIALS**

June 2025

When are people willing to donate their time or money after a disaster? We investigate the psychological and socio-economic determinants of post-disaster giving in Japan, using a nationally representative panel survey of more than 7,000 respondents, conducted repeatedly from early 2020, including after the 2024 Noto Peninsula earthquake. We examine how individual characteristics—including past disaster experience, social capital (trust, reciprocity, cooperation), ‘Big-5’ personality traits, and digital behaviours—influence the likelihood of engaging in various forms of post-disaster assistance, from traditional monetary donations to newer digitally-facilitated acts such as online shopping for Noto products. Our analysis finds that prior disaster experience and personal openness are consistent robust predictors of prosocial behaviour. The relationship between social capital and aid activities is more subtle. Trust and cooperation are both positively associated with post-disaster assistance, but this is not the case for reciprocity. These findings emphasise that nuanced conceptualisation of social capital is required and underscore the need for caution in assuming its universal relevance in mobilising disaster aid. We conclude by suggesting directions for future research that more precisely delineate the interplay between social, psychological, and socio-economic factors in shaping post-disaster giving.

**Keywords:** earthquake, post-disaster aid, social capital

Online Appendix 1: Figure 1

|  |  |  | donation | crowdfunding | hometown tax | online shopping | tourism (without subsidies) | tourism (with subsidies) | volunteer |
| --- | --- | --- | --- | --- | --- | --- | --- | --- | --- |
|  | 3 | Done several times | 753 | 274 | 468 | 295 | 221 | 191 | 209 |
|  | 2 | Done once | 1,315 | 461 | 532 | 491 | 414 | 412 | 342 |
|  | 1 | Will do soon | 987 | 803 | 1,405 | 1,406 | 1,316 | 1,227 | 710 |
|  | 0 | None | 7,615 | 9,132 | 8,265 | 8,478 | 8,719 | 8,840 | 9,409 |

Online Appendix 2: Full results for Table 1

| **Table: Ordered logit estimations: Dependent is frequency of each support** | | | | | | | | |  |  |  |  |  |  |  |  |  |  |  |  |  |
| --- | --- | --- | --- | --- | --- | --- | --- | --- | --- | --- | --- | --- | --- | --- | --- | --- | --- | --- | --- | --- | --- |
|  | **Donation** | |  | **Crowdfunding** | |  | **Hometown donation to Hokuriku** | |  | **Online shopping on Hokuriku products** | |  | **Tourism in Hokuriku (without subsidies)** | |  | **Tourism in Hokuriku (with subsidies)** | |  | **Disaster volunteer activities** | |  |
|  | **Coeff** |  |  | **Coeff** |  |  | **Coeff** |  |  | **Coeff** |  |  | **Coeff** |  |  | **Coeff** |  |  | **Coeff** |  |  |
| sex | 0.3665 | 5.62 | *** | 0.1749 | 1.51 |  | 0.20039 | 2.06 | ** | 0.181 | 1.86 | * | 0.0406 | 0.5 |  | 0.0212 | 0.23 |  | 0.0015 | 0.01 |  |
| income | 0.0167 | 1.66 | * | 0.0167 | 1 |  | 0.04596 | 3.94 | *** | 0.024 | 1.76 | * | 0.0126 | 1.23 |  | 0.0125 | 0.85 |  | 0.0259 | 1.48 |  |
| age | 0.0099 | 0.92 |  | -0.098 | -6.88 | *** | -0.0492 | -3.4 | *** | -0.01 | -0.58 |  | -0.061 | -3.41 | *** | -0.073 | -4.49 | *** | -0.14 | -7.7 | *** |
| risk | 0.0687 | 7.55 | *** | 0.1309 | 8.48 | *** | 0.09707 | 6.82 | *** | 0.094 | 7.64 | *** | 0.1202 | 6.79 | *** | 0.1156 | 7.29 | *** | 0.1712 | 9.19 | *** |
| education | 0.1917 | 6.77 | *** | 0.173 | 4.36 | *** | 0.22385 | 5.82 | *** | 0.205 | 4.65 | *** | 0.1783 | 4.27 | *** | 0.1597 | 4.77 | *** | 0.1527 | 3.17 | *** |
| Past disaster exp | 1.0418 | 9.96 | *** | 1.6185 | 13.67 | *** | 1.3699 | 11.56 | *** | 1.392 | 9.5 | *** | 1.4972 | 9.27 | *** | 1.4604 | 9.53 | *** | 1.6426 | 9.87 | *** |
| Extraversion | -0.0028 | -0.13 |  | 0.0508 | 1.36 |  | 0.07124 | 2.15 | ** | 0.043 | 1.47 |  | 0.0925 | 2.83 | *** | 0.085 | 2.8 | ** | 0.0917 | 2.42 | ** |
| Agreeableness | 0.0378 | 1.61 | * | -3E-04 | -0.01 |  | 0.01003 | 0.37 |  | 0.004 | 0.19 |  | -0.005 | -0.16 |  | 0.0202 | 0.56 |  | 0.0064 | 0.18 |  |
| Conscientiousness | 0.0412 | 1.5 |  | 0.0254 | 0.64 |  | 0.05252 | 1.76 | * | 0.033 | 1.2 |  | 0.0428 | 1.63 | * | 0.0427 | 1.52 |  | 0.028 | 0.57 |  |
| Neuroticism | 0.0186 | 0.7 |  | -0.01 | -0.39 |  | 0.04171 | 1.63 | * | -0.04 | -1.16 |  | -0.039 | -1.4 |  | 0.0189 | 0.77 |  | -0.002 | -0.06 |  |
| Openness | 0.1244 | 4.53 | *** | 0.2421 | 6.34 | *** | 0.11118 | 4.01 | *** | 0.162 | 7.57 | *** | 0.0918 | 2.45 | ** | 0.1678 | 3.71 | *** | 0.2235 | 5.48 | *** |
| trust | 0.0225 | 0.58 |  | 0.1985 | 2.82 | *** | 0.19229 | 3.44 | *** | 0.113 | 2.56 | ** | 0.0834 | 1.39 |  | 0.0695 | 1.22 |  | 0.2414 | 4.12 | *** |
| reciprocity | 0.1767 | 4.88 | *** | -0.201 | -3.32 | *** | -0.0062 | -0.1 |  | -0.01 | -0.14 |  | -0.009 | -0.13 |  | -0.06 | -0.71 |  | -0.196 | -3.33 | *** |
| cooperation | 0.2972 | 6.6 | ** | 0.0181 | 0.28 |  | 0.10023 | 2.2 | ** | 0.238 | 6.15 | *** | 0.1153 | 2.37 | ** | 0.1262 | 2.34 | ** | -0.181 | -3.81 | *** |
| Nob | 7,194 |  |  | 7,194 |  |  | 7,194 |  |  | 7,194 |  |  | 7,194 |  |  | 7,194 |  |  | 7,194 |  |  |
| Log pseudolikelihood | -6081.7 |  |  | -3501 |  |  | -4982.8 |  |  | -4571 |  |  | -4053 |  |  | -3866 |  |  | -2826 |  |  |
| job dummies, pref dummies are omitted | | | | |  |  |  |  |  |  |  |  |  |  |  |  |  |  |  |  |  |
| Clustered errors (pref) | |  |  |  |  |  |  |  |  |  |  |  |  |  |  |  |  |  |  |  |  |

Online Appendix 3: Logit estimations

| **Logit estimations** | | |  |  |  |  |  |  |  |  |  |  |  |  |  |  |  |  |  |  |  |
| --- | --- | --- | --- | --- | --- | --- | --- | --- | --- | --- | --- | --- | --- | --- | --- | --- | --- | --- | --- | --- | --- |
|  | **Donation** | |  | **Crowdfunding** | |  | **Hometown donation to Hokuriku** | |  | **Online shopping on Hokuriku products** | |  | **Tourism in Hokuriku (without subsidies)** | |  | **Tourism in Hokuriku (with subsidies)** | |  | **Disaster volunteer activities** | |  |
| sex | 0.362 | 5.25 | *** | 0.15973 | 1.38 |  | 0.18467 | 1.83 | * | 0.1555 | 1.69 | * | 0.0152 | 0.19 |  | -0.012 | -0.13 |  | -0.0252 | -0.22 |  |
| income | 0.0119 | 1.05 |  | 0.01124 | 0.71 |  | 0.04037 | 3.43 | *** | 0.0173 | 1.4 |  | 0.0102 | 0.96 |  | 0.0105 | 0.74 |  | 0.01987 | 1.16 |  |
| age | 0.0104 | 1.05 |  | -0.0954 | -6.77 | *** | -0.0408 | -2.96 | *** | 0.0003 | 0.02 |  | -0.054 | -3.04 | *** | -0.064 | -4.07 | *** | -0.1351 | -7.76 | *** |
| risk | 0.0787 | 9.02 | *** | 0.13109 | 8.52 | *** | 0.09822 | 6.88 | *** | 0.0914 | 7 | *** | 0.1146 | 6.63 | *** | 0.1096 | 6.89 | *** | 0.16824 | 9.41 | *** |
| education | 0.1948 | 6.99 | *** | 0.17959 | 4.46 | *** | 0.23001 | 6.1 | *** | 0.2035 | 4.59 | *** | 0.182 | 4.48 | *** | 0.1547 | 4.74 | *** | 0.14088 | 2.98 | *** |
| past disaster exp | 1.1964 | 9.15 | *** | 1.5972 | 12.61 | *** | 1.30645 | 9.93 | *** | 1.3458 | 9.05 | *** | 1.4139 | 8.64 | *** | 1.3412 | 9.03 | *** | 1.56579 | 9.06 | *** |
| Extraversion | 0.0024 | 0.11 |  | 0.05734 | 1.57 |  | 0.06261 | 1.94 | * | 0.035 | 1.15 |  | 0.0906 | 2.85 | *** | 0.0796 | 2.7 | ** | 0.09823 | 2.56 | ** |
| Agreeableness | 0.0458 | 2.02 | ** | 0.00558 | 0.14 |  | 0.01208 | 0.44 |  | 0.0121 | 0.55 |  | 0.004 | 0.13 |  | 0.026 | 0.76 |  | 0.00831 | 0.24 |  |
| Conscientiousness | 0.0481 | 1.93 | * | 0.02032 | 0.52 |  | 0.04328 | 1.43 |  | 0.0277 | 1.04 |  | 0.042 | 1.49 |  | 0.0498 | 1.82 | * | 0.02906 | 0.59 |  |
| Neuroticism | 0.0411 | 1.51 |  | 0.00448 | 0.18 |  | 0.05341 | 1.79 |  | -0.03 | -0.89 |  | -0.028 | -0.97 |  | 0.038 | 1.56 |  | 0.01797 | 0.52 |  |
| Openness | 0.1126 | 3.53 | *** | 0.2328 | 6.15 | *** | 0.11053 | 4.02 | *** | 0.1604 | 7.32 | *** | 0.0986 | 2.64 | ** | 0.1768 | 3.87 | *** | 0.22437 | 4.95 | *** |
| trust | 0.0518 | 1.43 |  | 0.20391 | 2.75 | *** | 0.19409 | 3.25 | *** | 0.1123 | 2.64 | ** | 0.0754 | 1.28 |  | 0.0586 | 1.04 |  | 0.23954 | 4.16 | *** |
| reciprocity | 0.1359 | 3.48 | *** | -0.2085 | -3.07 | *** | -0.0164 | -0.24 |  | -0.009 | -0.14 |  | 0.008 | 0.12 |  | -0.043 | -0.51 |  | -0.195 | -3.42 | *** |
| cooperation | 0.2976 | 6.92 | *** | 0.0298 | 0.51 |  | 0.13401 | 3.01 | *** | 0.2579 | 6.55 | *** | 0.1346 | 2.99 | *** | 0.1412 | 2.67 | ** | -0.1616 | -3.42 | *** |
| Nob | 7,194 |  |  | 7,158 |  |  | 7,194 |  |  | 7,194 |  |  | 7,158 |  |  | 7,177 |  |  | 7,067 |  |  |
| Log pseudolikelihood | -3962 |  |  | -2547.7 |  |  | -3469.6 |  |  | -3328 |  |  | -3051 |  |  | -2935 |  |  | -2113.4 |  |  |
| job dummies, pref dummies are omitted | | | | |  |  |  |  |  |  |  |  |  |  |  |  |  |  |  |  |  |
| Clustered errors (pref) | |  |  |  |  |  |  |  |  |  |  |  |  |  |  |  |  |  |  |  |  |

Online Appendix 4: Full results for Table 2

|  | Donation | Crowdfunding | Hometown donation | Online shopping | Tourism | Tourism | Volunteer |
| --- | --- | --- | --- | --- | --- | --- | --- |
|  |  |  |  |  |  |  |  |
| sex | 0.344*** | 0.116 | 0.161* | 0.143 | 0.00822 | -0.00456 | -0.05 |
|  | (5.27) | (1.17) | (1.78) | (1.52) | (0.11) | (-0.06) | (-0.48) |
|  |  |  |  |  |  |  |  |
| income | 0.00248 | -0.00458 | 0.0304*** | 0.00706 | -0.00766 | -0.0111 | 0.00756 |
|  | (0.26) | (-0.27) | (2.79) | (0.53) | (-0.75) | (-0.73) | (0.40) |
|  |  |  |  |  |  |  |  |
| age | 0.0308*** | -0.051*** | -0.0202 | 0.0382*** | -0.0246 | -0.0332** | -0.087*** |
|  | (2.89) | (-4.40) | (-1.43) | (3.53) | (-1.51) | (-2.30) | (-4.66) |
|  |  |  |  |  |  |  |  |
| risk | 0.0527*** | 0.0930*** | 0.0710*** | 0.0665*** | 0.0934*** | 0.0822*** | 0.135*** |
|  | (4.97) | (5.26) | (4.32) | (5.17) | (5.19) | (5.02) | (6.07) |
|  |  |  |  |  |  |  |  |
| education | 0.164*** | 0.117*** | 0.176*** | 0.162*** | 0.125*** | 0.102*** | 0.0983** |
|  | (5.65) | (3.05) | (4.80) | (3.73) | (2.91) | (2.83) | (2.08) |
|  |  |  |  |  |  |  |  |
| past disaster | 0.804*** | 1.241*** | 1.082*** | 1.092*** | 1.158*** | 1.073*** | 1.190*** |
|  | (7.36) | (12.01) | (10.16) | (8.56) | (7.39) | (7.37) | (7.19) |
|  |  |  |  |  |  |  |  |
| Extraversion | -0.0144 | 0.0121 | 0.0535* | 0.0219 | 0.0669** | 0.0531* | 0.0437 |
|  | (-0.64) | (0.36) | (1.74) | (0.86) | (2.11) | (1.75) | (1.15) |
|  |  |  |  |  |  |  |  |
| Agreeableness | 0.0482* | 0.0102 | 0.02 | 0.0177 | 0.00421 | 0.0346 | 0.013 |
|  | (1.96) | (0.23) | (0.76) | (0.73) | (0.12) | (0.85) | (0.33) |
|  |  |  |  |  |  |  |  |
| Conscientiousness | 0.0431 | 0.0454 | 0.0589* | 0.0327 | 0.0432 | 0.0466* | 0.0392 |
|  | (1.52) | (1.14) | (1.88) | (1.19) | (1.56) | (1.70) | (0.70) |
|  |  |  |  |  |  |  |  |
| Neuroticism | 0.0333 | 0.0273 | 0.0681*** | -0.012 | -0.0224 | 0.0457 | 0.038 |
|  | (1.15) | (0.83) | (2.89) | (-0.34) | (-0.78) | (1.63) | (1.11) |
|  |  |  |  |  |  |  |  |
| Openness | 0.0926*** | 0.200*** | 0.0683** | 0.109*** | 0.0403 | 0.123** | 0.189*** |
|  | (3.49) | (5.12) | (2.36) | (4.80) | (1.06) | (2.57) | (4.17) |
|  |  |  |  |  |  |  |  |
| trust | 0.0149 | 0.168** | 0.182*** | 0.0924* | 0.0682 | 0.0405 | 0.227*** |
|  | (0.35) | (2.25) | (3.03) | (1.94) | (1.10) | (0.65) | (4.05) |
|  |  |  |  |  |  |  |  |
| reciprocity | 0.171*** | -0.168** | 0.00273 | -0.00395 | -0.00554 | -0.0326 | -0.139** |
|  | (4.49) | (-2.33) | (0.04) | (-0.06) | (-0.08) | (-0.36) | (-2.30) |
|  |  |  |  |  |  |  |  |
| cooperation | 0.278*** | 0.0185 | 0.0793 | 0.235*** | 0.108** | 0.136** | -0.176*** |
|  | (6.30) | (0.25) | (1.54) | (4.98) | (1.99) | (1.97) | (-3.67) |
|  |  |  |  |  |  |  |  |
| Digital payments | 0.283*** | 0.131* | 0.404*** | 0.168*** | 0.320*** | 0.280*** | 0.0149 |
|  | (4.16) | (1.91) | (7.07) | (3.64) | (5.58) | (5.31) | (0.26) |
|  |  |  |  |  |  |  |  |
| Cash payments | 0.127*** | -0.124*** | -0.0911** | -0.0692** | -0.0339 | -0.115*** | -0.127*** |
|  | (3.82) | (-3.22) | (-2.44) | (-2.20) | (-0.99) | (-2.94) | (-2.87) |
|  |  |  |  |  |  |  |  |
| Share economy | 0.410*** | 0.783*** | 0.550*** | 0.649*** | 0.709*** | 0.832*** | 0.902*** |
|  | (8.64) | (11.11) | (8.39) | (8.40) | (12.48) | (11.54) | (14.06) |
|  |  |  |  |  |  |  |  |
| Online shopping | 0.399*** | 0.494*** | 0.367*** | 0.599*** | 0.347*** | 0.324*** | 0.350*** |
|  | (9.85) | (7.80) | (6.65) | (9.18) | (5.36) | (5.10) | (4.85) |
|  |  |  |  |  |  |  |  |
| Log pseudolikelihood | -5864.36 | -3224.36 | -4713.82 | -4255.45 | -3780.68 | -3559.68 | -2594.91 |
| nob | 7,194 | 7,194 | 7,194 | 7,194 | 7,194 | 7,194 | 7,194 |

Online Appendix 5: Full results for Table 3

|  | Donation | Crowdfunding | Hometown donation | Online shopping | Tourism | Tourism | Volunteer |
| --- | --- | --- | --- | --- | --- | --- | --- |
|  |  |  |  |  |  |  |  |
| sex | 0.455*** | 0.273** | 0.295*** | 0.267*** | 0.131 | 0.122 | 0.113 |
|  | (6.94) | (2.37) | (3.00) | (2.80) | (1.64) | (1.35) | (1.01) |
|  |  |  |  |  |  |  |  |
| income | -0.0005 | 0.00118 | 0.0287** | 0.00578 | -0.00484 | -0.00659 | 0.0106 |
|  | (-0.05) | (0.07) | (2.55) | (0.44) | (-0.48) | (-0.47) | (0.62) |
|  |  |  |  |  |  |  |  |
| age | 0.0209* | -0.0852*** | -0.0417*** | 0.00578 | -0.0508*** | -0.061*** | -0.12*** |
|  | (1.70) | (-5.90) | (-2.94) | (0.46) | (-2.78) | (-3.78) | (-6.96) |
|  |  |  |  |  |  |  |  |
| risk | 0.0547*** | 0.114*** | 0.0846*** | 0.0789*** | 0.108*** | 0.102*** | 0.156*** |
|  | (5.85) | (7.29) | (5.69) | (6.53) | (6.19) | (6.97) | (8.04) |
|  |  |  |  |  |  |  |  |
| education | 0.148*** | 0.137*** | 0.172*** | 0.165*** | 0.132*** | 0.113*** | 0.109** |
|  | (5.10) | (3.51) | (4.36) | (3.61) | (3.07) | (3.11) | (2.18) |
|  |  |  |  |  |  |  |  |
| past disaster | 0.943*** | 1.493*** | 1.264*** | 1.283*** | 1.387*** | 1.347*** | 1.489*** |
|  | (9.30) | (13.17) | (10.96) | (9.35) | (8.86) | (9.01) | (9.42) |
|  |  |  |  |  |  |  |  |
| Extraversion | -0.0114 | 0.0406 | 0.0625* | 0.0359 | 0.0832*** | 0.0767*** | 0.0793** |
|  | (-0.53) | (1.07) | (1.88) | (1.18) | (2.62) | (2.59) | (2.20) |
|  |  |  |  |  |  |  |  |
| Agreeableness | 0.0506** | 0.00828 | 0.023 | 0.0158 | 0.0041 | 0.0311 | 0.0125 |
|  | (2.40) | (0.22) | (0.95) | (0.72) | (0.13) | (0.92) | (0.38) |
|  |  |  |  |  |  |  |  |
| Conscientiousness | 0.0293 | 0.015 | 0.043 | 0.0178 | 0.0319 | 0.032 | 0.018 |
|  | (1.04) | (0.37) | (1.41) | (0.66) | (1.24) | (1.18) | (0.37) |
|  |  |  |  |  |  |  |  |
| Neuroticism | 0.0126 | -0.0166 | 0.0416 | -0.0408 | -0.0406 | 0.0149 | -0.00782 |
|  | (0.49) | (-0.65) | (1.55) | (-1.23) | (-1.50) | (0.59) | (-0.21) |
|  |  |  |  |  |  |  |  |
| Openness | 0.103*** | 0.214*** | 0.0836*** | 0.130*** | 0.0639 | 0.142*** | 0.202*** |
|  | (4.11) | (5.80) | (3.09) | (6.10) | (1.62) | (3.14) | (4.40) |
|  |  |  |  |  |  |  |  |
| trust | 0.0285 | 0.211*** | 0.205*** | 0.118*** | 0.0976* | 0.0852 | 0.261*** |
|  | (0.74) | (2.97) | (3.67) | (2.71) | (1.67) | (1.54) | (4.50) |
|  |  |  |  |  |  |  |  |
| reciprocity | 0.149*** | -0.236*** | -0.0441 | -0.044 | -0.051 | -0.101 | -0.23*** |
|  | (3.98) | (-3.87) | (-0.68) | (-0.71) | (-0.76) | (-1.17) | (-3.94) |
|  |  |  |  |  |  |  |  |
| cooperation | 0.284*** | 0.00641 | 0.0827* | 0.220*** | 0.101** | 0.112** | -0.18*** |
|  | (6.33) | (0.09) | (1.77) | (5.94) | (2.11) | (2.21) | (-3.94) |
| Routine | -0.0940** | -0.113** | -0.0856** | -0.198*** | -0.121*** | -0.114** | -0.0103 |
|  | (-2.57) | (-2.39) | (-2.15) | (-4.37) | (-2.58) | (-2.50) | (-0.17) |
|  |  |  |  |  |  |  |  |
| Abstract | 0.251*** | 0.227*** | 0.307*** | 0.207*** | 0.257*** | 0.275*** | 0.319*** |
|  | (6.33) | (4.35) | (7.40) | (5.37) | (5.36) | (5.99) | (6.53) |
|  |  |  |  |  |  |  |  |
| Manual | 0.106*** | 0.198*** | 0.0656* | 0.110*** | 0.119*** | 0.148*** | 0.228*** |
|  | (2.85) | (4.39) | (1.75) | (2.98) | (4.17) | (3.98) | (4.48) |
| Log pseudolikelihood | -6004.9 | -3435.94 | -4908.07 | -4488.9 | -3984.48 | -3790.58 | -2767.28 |
| nob | 7,194 | 7,194 | 7,194 | 7,194 | 7,194 | 7,194 | 7,194 |

Online Appendix 6: Working environments

|  | Donation | Crowdfunding | Hometown donation | Online shopping | Tourism | Tourism | Volunteer |
| --- | --- | --- | --- | --- | --- | --- | --- |
|  |  |  |  |  |  |  |  |
| sex | 0.377*** | 0.217* | 0.224** | 0.203** | 0.0585 | 0.0533 | 0.0458 |
|  | (5.89) | (1.88) | (2.30) | (2.08) | (0.71) | (0.60) | (0.41) |
|  |  |  |  |  |  |  |  |
| income | 0.0149 | 0.0138 | 0.0447*** | 0.0218 | 0.00979 | 0.00925 | 0.023 |
|  | (1.49) | (0.82) | (3.84) | (1.58) | (0.92) | (0.63) | (1.32) |
|  |  |  |  |  |  |  |  |
| age | 0.01 | -0.094*** | -0.046*** | -0.00474 | -0.059*** | -0.069*** | -0.135*** |
|  | (0.96) | (-7.05) | (-3.32) | (-0.39) | (-3.45) | (-4.52) | (-7.77) |
|  |  |  |  |  |  |  |  |
| risk | 0.0676*** | 0.124*** | 0.0932*** | 0.0892*** | 0.117*** | 0.110*** | 0.164*** |
|  | (7.39) | (8.23) | (6.69) | (7.25) | (6.70) | (6.88) | (8.49) |
|  |  |  |  |  |  |  |  |
| education | 0.181*** | 0.163*** | 0.217*** | 0.195*** | 0.166*** | 0.147*** | 0.146*** |
|  | (6.49) | (4.10) | (5.57) | (4.43) | (3.90) | (4.28) | (2.98) |
|  |  |  |  |  |  |  |  |
| past disaster | 1.057*** | 1.597*** | 1.355*** | 1.390*** | 1.507*** | 1.448*** | 1.609*** |
|  | (10.13) | (13.37) | (11.64) | (9.69) | (9.36) | (9.65) | (9.62) |
|  |  |  |  |  |  |  |  |
| Extraversion | -0.00426 | 0.043 | 0.0689** | 0.0396 | 0.0895*** | 0.0801*** | 0.0849** |
|  | (-0.20) | (1.17) | (2.09) | (1.35) | (2.81) | (2.65) | (2.18) |
|  |  |  |  |  |  |  |  |
| Agreeableness | 0.0390* | 0.00558 | 0.0134 | 0.00744 | -0.00302 | 0.0243 | 0.0133 |
|  | (1.69) | (0.14) | (0.51) | (0.32) | (-0.09) | (0.68) | (0.37) |
|  |  |  |  |  |  |  |  |
| Conscientiousness | 0.0406 | 0.0224 | 0.0501 | 0.0298 | 0.0404 | 0.0398 | 0.0255 |
|  | (1.47) | (0.56) | (1.63) | (1.08) | (1.52) | (1.38) | (0.49) |
|  |  |  |  |  |  |  |  |
| Neuroticism | 0.0235 | -0.0157 | 0.0401 | -0.0365 | -0.035 | 0.018 | -0.0113 |
|  | (0.91) | (-0.59) | (1.51) | (-1.10) | (-1.25) | (0.67) | (-0.28) |
|  |  |  |  |  |  |  |  |
| Openness | 0.119*** | 0.232*** | 0.103*** | 0.154*** | 0.0842** | 0.158*** | 0.215*** |
|  | (4.39) | (5.79) | (3.89) | (7.53) | (2.24) | (3.41) | (4.97) |
|  |  |  |  |  |  |  |  |
| trust | 0.00769 | 0.183** | 0.183*** | 0.100** | 0.0694 | 0.0537 | 0.230*** |
|  | (0.20) | (2.52) | (3.24) | (2.17) | (1.13) | (0.90) | (3.72) |
|  |  |  |  |  |  |  |  |
| reciprocity | 0.160*** | -0.196*** | -0.00896 | -0.0156 | -0.0227 | -0.0651 | -0.180*** |
|  | (4.37) | (-3.22) | (-0.14) | (-0.24) | (-0.33) | (-0.77) | (-3.04) |
|  |  |  |  |  |  |  |  |
| cooperation | 0.283*** | 0.0109 | 0.0913** | 0.226*** | 0.1000** | 0.115** | -0.192*** |
|  | (6.20) | (0.16) | (2.00) | (5.76) | (2.15) | (2.23) | (-3.95) |
|  |  |  |  |  |  |  |  |
| Teamwork base | 0.0666* | -0.134*** | -0.0102 | 0.00851 | 0.0344 | -0.0453 | -0.219*** |
|  | (1.86) | (-2.61) | (-0.30) | (0.28) | (0.86) | (-1.14) | (-4.34) |
|  |  |  |  |  |  |  |  |
| Outcome base wage | 0.0241 | 0.251*** | 0.151*** | 0.124** | 0.0929** | 0.194*** | 0.292*** |
|  | (0.78) | (5.13) | (3.56) | (2.14) | (2.35) | (4.84) | (6.54) |
|  |  |  |  |  |  |  |  |
| Flexible time | 0.0962** | 0.118** | 0.0279 | 0.0734 | 0.0962** | 0.102** | 0.112** |
|  | (2.32) | (2.52) | (0.89) | (1.53) | (2.11) | (2.27) | (2.12) |
|  |  |  |  |  |  |  |  |
| Log pseudolikelihood | -6068.75 | -3477.36 | -4971.48 | -4558.41 | -4040.59 | -3846.2 | -2803.14 |
| nob | 7,194 | 7,194 | 7,194 | 7,194 | 7,194 | 7,194 | 7,194 |

Online Appendix 7: Geographical proximity

|  |  |  |  |  |  |  |  |
| --- | --- | --- | --- | --- | --- | --- | --- |
|  | Donation | Crowdfunding | Hometown donation | Online shopping | Tourism | Tourism | Volunteer |
|  |  |  |  |  |  |  |  |
| sex | 0.355*** | 0.168 | 0.193** | 0.183* | 0.0403 | 0.0198 | -0.0050 |
|  | (5.55) | (1.51) | (2.03) | (1.94) | (0.50) | (0.23) | (-0.05) |
|  |  |  |  |  |  |  |  |
| income | 0.015 | 0.0158 | 0.0452*** | 0.0246** | 0.0132 | 0.0126 | 0.0235 |
|  | (1.55) | (0.98) | (4.11) | (1.99) | (1.31) | (0.89) | (1.36) |
|  |  |  |  |  |  |  |  |
| age | 0.00889 | -0.101*** | -0.051*** | -0.0102 | -0.064*** | -0.074*** | -0.14*** |
|  | (0.84) | (-7.16) | (-3.61) | (-0.82) | (-3.58) | (-4.59) | (-7.81) |
|  |  |  |  |  |  |  |  |
| risk | 0.0697*** | 0.130*** | 0.0968*** | 0.0946*** | 0.119*** | 0.115*** | 0.171*** |
|  | (7.51) | (8.56) | (6.96) | (7.73) | (6.81) | (7.17) | (9.15) |
|  |  |  |  |  |  |  |  |
| education | 0.181*** | 0.164*** | 0.226*** | 0.208*** | 0.177*** | 0.158*** | 0.133*** |
|  | (6.46) | (4.03) | (5.88) | (4.55) | (4.27) | (4.81) | (2.72) |
|  |  |  |  |  |  |  |  |
| past disaster | 1.039*** | 1.632*** | 1.392*** | 1.417*** | 1.520*** | 1.480*** | 1.666*** |
|  | (9.90) | (14.08) | (12.17) | (10.32) | (10.21) | (10.24) | (10.33) |
|  |  |  |  |  |  |  |  |
| Extraversion | -0.00432 | 0.049 | 0.0754** | 0.0469* | 0.0915*** | 0.0849*** | 0.0921** |
|  | (-0.20) | (1.37) | (2.30) | (1.67) | (2.80) | (2.79) | (2.45) |
|  |  |  |  |  |  |  |  |
| Agreeableness | 0.0299 | -0.00428 | 0.00729 | 0.00405 | -0.00588 | 0.0181 | -0.0021 |
|  | (1.26) | (-0.10) | (0.27) | (0.17) | (-0.19) | (0.51) | (-0.06) |
|  |  |  |  |  |  |  |  |
| Conscientiousness | 0.0367 | 0.0232 | 0.0503* | 0.0308 | 0.0439* | 0.0437 | 0.0244 |
|  | (1.32) | (0.58) | (1.72) | (1.14) | (1.65) | (1.52) | (0.50) |
|  |  |  |  |  |  |  |  |
| Neuroticism | 0.0145 | -0.0135 | 0.0389 | -0.0411 | -0.038 | 0.0153 | -0.0042 |
|  | (0.54) | (-0.53) | (1.50) | (-1.33) | (-1.37) | (0.63) | (-0.12) |
|  |  |  |  |  |  |  |  |
| Openness | 0.123*** | 0.242*** | 0.110*** | 0.161*** | 0.0932** | 0.168*** | 0.219*** |
|  | (4.45) | (6.45) | (3.98) | (7.54) | (2.56) | (3.75) | (5.34) |
|  |  |  |  |  |  |  |  |
| trust | 0.0313 | 0.203*** | 0.189*** | 0.112** | 0.0838 | 0.0677 | 0.254*** |
|  | (0.79) | (2.87) | (3.35) | (2.49) | (1.41) | (1.19) | (4.36) |
|  |  |  |  |  |  |  |  |
| reciprocity | 0.177*** | -0.197*** | -0.00215 | -0.00785 | -0.0064 | -0.0534 | -0.20*** |
|  | (4.96) | (-3.25) | (-0.03) | (-0.13) | (-0.09) | (-0.63) | (-3.44) |
|  |  |  |  |  |  |  |  |
| cooperation | 0.297*** | 0.0191 | 0.102** | 0.243*** | 0.111** | 0.126** | -0.17*** |
|  | (6.58) | (0.29) | (2.19) | (6.23) | (2.30) | (2.33) | (-3.78) |
|  |  |  |  |  |  |  |  |
| ln_distance | -0.0169 | -0.109 | -0.076 | -0.0815 | -0.428*** | -0.418*** | -0.200** |
|  | (-0.22) | (-1.64) | (-1.40) | (-1.40) | (-7.41) | (-5.83) | (-2.33) |
|  |  |  |  |  |  |  |  |
| Log pseudolikelihood | -6121.45 | -3515.85 | -5001.07 | -4588.97 | -4070.68 | -3888.52 | -2842.5 |
| nob | 7,194 | 7,194 | 7,194 | 7,194 | 7,194 | 7,194 | 7,194 |
